# FMCW Radar Micro-Doppler UAV Classification via Compact Depthwise CNN and Offline Temporal Logit Fusion

**DOI:** 10.3390/s26206656

**Published:** 2026-10-21

**Authors:** Haida Yang, Qiang Lin, Yu Yao, Lijuan Wang

**Affiliations:** Air-Defense Early Warning Equipment Department, Air Force Early Warning Academy, Wuhan 430019, China; linqiang_wuhan@163.com (Q.L.); 2000yaoyu@163.com (Y.Y.); lijuanwang027@163.com (L.W.)

**Keywords:** FMCW radar, micro-Doppler, UAV classification, depthwise CNN, temporal logit fusion, recording-disjoint evaluation

## Abstract

Frequency-modulated continuous-wave (FMCW) radar micro-Doppler signatures provide informative motion cues for small unmanned aerial vehicle (UAV) classification, but independent single-window predictions can vary across neighboring time windows. This study evaluates a compact scratch-trained depthwise convolutional neural network (CNN) with a deterministic three-channel observation interface and a parameter-free offline temporal logit fusion stage under a recording-disjoint six-class protocol. The classifier contains 52,918 trainable parameters; temporal fusion is applied only as final post-processing, without retraining or increasing model size. Across three recording-disjoint folds and three model seeds, the proposed three-channel single-window setting achieved 98.35±0.90% macro-F1, compared with 97.67% for a repeated-channel reference. Applying short-neighborhood fusion with K=3 further increased mean macro-F1 to 98.60%. A seven-configuration per-channel ablation shows that no single channel accounts for this gain, and an expanded subset with varied modulation periods, frequency bands, and nominal ranges provides initial evidence on sensitivity to radar acquisition parameters. These results indicate that the proposed observation interface improves over simple channel repetition, while offline temporal aggregation provides a small additional gain for the compact classifier within the evaluated held-out-recording protocol.

## 1. Introduction

Small unmanned aerial vehicles (UAVs) have become increasingly relevant to low-altitude sensing because differences in platform size, rotor configuration, and payload capability can affect monitoring and assessment requirements [1,2,3]. In this setting, classification is distinct from target detection or range localization: it assigns a known target type to radar observations after a relevant target return has been obtained. This study focuses on six-class UAV classification under a recording-disjoint evaluation protocol.

Radar measurements can provide range- and motion-related information without relying on visible illumination. Frequency-modulated continuous-wave (FMCW) radar provides range-resolved measurements together with Doppler information, and returns from rotary-wing UAVs can contain micro-Doppler components associated with blade rotation, blade flashes, body motion, and maneuvering [4,5,6,7]. These time-varying signatures make time–frequency micro-Doppler observations a natural basis for UAV classification.

Prior radar studies have used micro-Doppler signatures and spectrogram-like observations for UAV classification and related discrimination tasks involving small airborne targets [8,9,10,11,12]. However, the interpretation of window-based classification performance depends not only on the classifier, but also on how observations are constructed, how temporal evidence is used, and how train–test partitions are formed. In short-time windows, predictions may fluctuate because of local changes in aspect, blade visibility, signal strength, or maneuvering.

Compact convolutional neural networks (CNNs) provide a practical way to learn discriminative structure from time–frequency radar observations while keeping model size limited. Lightweight CNN-based approaches have been explored in radar and UAV recognition settings [13,14,15,16]. In this study, a scratch-trained depthwise CNN is used as a compact window-level classifier, and a repeated-channel input is included as a reference for assessing the proposed three-channel observation interface.

Temporal context provides an additional source of evidence for window-based classification. Adjacent windows within the same recording may contain complementary information about the same UAV, whereas independent single-window decisions can vary locally. Although prior radar work has considered temporal modeling or multi-representation evidence combination in related settings [17,18,19,20], the approach evaluated here is deliberately simpler: the trained single-window classifier is kept fixed, and temporal evidence is incorporated only through temporal logit fusion as final post-processing.

Evaluation design is also important when long radar recordings are divided into many windows. Windows from the same source recording can share trajectory, acquisition conditions, background structure, and raw signal content. Randomly distributing such windows across training and test partitions can therefore create a leakage risk and produce optimistic estimates for a held-out-recording question [21]. To reduce this overlap, this study groups data by source recording so that no source recording contributes windows to both training and testing within a fold. This design prevents direct source-recording overlap, while not controlling for all acquisition-session, nominal-range, geometry, or other condition-related variations.

Against this background, this study evaluates whether a deterministic three-channel micro-Doppler observation interface and a simple temporal decision stage can improve a compact FMCW radar UAV classifier under recording-disjoint evaluation. The proposed observation interface and depthwise CNN provide window-level logits, while temporal context is introduced only at the final decision stage through logit fusion.

Accordingly, this study makes the following contributions:

- It evaluates a compact single-window FMCW radar UAV classifier for six known UAV types using a deterministic three-channel micro-Doppler observation interface and a scratch-trained depthwise CNN under recording-disjoint evaluation.
- It includes a repeated-channel input as a component-level reference for the proposed observation interface and compares it with the matched single-window and fused settings, and it examines per-channel attribution through a seven-configuration ablation that zeroes individual input channels while keeping the classifier architecture and training protocol unchanged.
- It evaluates offline, centered, within-recording temporal logit fusion as final post-processing, and characterizes sensitivity to temporal-neighborhood length across recording-disjoint folds and multiple model seeds.
- It provides initial evidence on sensitivity to radar acquisition parameters by applying the same architecture and protocol to an expanded subset of recordings with varied modulation periods, frequency bands, and nominal ranges.

The remainder of this paper is organized as follows. Section 2 reviews related work in radar-based UAV classification, time–frequency observation design, compact classifiers, temporal evidence aggregation, and recording-disjoint evaluation. Section 3 describes the dataset, signal-processing pipeline, observation interface, classifier, temporal-fusion method, and evaluation protocol. Section 4 presents the experimental results, Section 5 discusses their interpretation and limitations, and Section 6 concludes the study.

## 2. Related Work

### 2.1. FMCW Radar and Micro-Doppler UAV Classification

Micro-Doppler analysis describes Doppler modulations induced by rotating, vibrating, or articulated target motions superimposed on bulk translational motion [4]. For rotary-wing UAVs, blade motion can produce blade flashes, propeller-rotation modulation lines, and related line-spectrum structures whose appearance may depend on target dynamics and sensing geometry [5,6,7]. These phenomena provide discriminative signal-domain cues for distinguishing UAV types, although their measured appearance remains conditional on the acquisition setting.

FMCW and other radar modalities have been used for UAV classification from micro-Doppler measurements. Oh et al. studied an FMCW-radar UAV classification system based on micro-Doppler signatures [8]; Park et al. investigated spectrogram-based UAV classification with CNNs [9]; and Leonardi et al. used multifunction radar measurements and micro-Doppler analysis for drone classification [10]. Sparse-reconstruction approaches have also been explored for UAV micro-Doppler signature detection and recognition [22], while radar studies of birds and small drones provide related evidence for discriminating small airborne targets [11,23]. Chen et al. introduced the LSS-FMCWR-1.0 dataset used in this study as a multiband FMCW radar dataset for low-slow-small target detection, together with a high-resolution micromotion feature-extraction method [24].

Detection and localization constitute distinct stages of the radar sensing chain and have been addressed separately in prior work [25,26]. Because the present classification pipeline assumes a range-localized target observation, the reported metrics characterize post-localization classification performance rather than end-to-end detection or localization performance.

### 2.2. Time–Frequency Observation Design for Radar Classification

Time–frequency representations connect radar micro-motion analysis with convolutional classification by organizing Doppler-frequency content over slow time. Spectrogram-based UAV classification has been investigated directly [9], and comparative work has examined how different micro-Doppler signal representations affect deep learning classification [27]. Other studies have proposed time–frequency processing to emphasize micro-motion features or have analyzed input formats for CNN processing of radar micro-Doppler signatures [28,29]. Together, these studies highlight the importance of explicitly defining the observation interface used for radar classification.

Multi-representation and multi-feature approaches further indicate that radar classifiers can operate on different representations of the measured signal [16,19,20,30]. In the present study, this motivation is used to define a deterministic three-channel micro-Doppler observation interface rather than to conduct a broad representation search. A repeated-channel input is included as a component-level reference for contextualizing the proposed observation interface.

### 2.3. Compact CNNs for Radar Sensing

Radar classification has also driven the development of compact neural architectures. Lightweight multiscale CNNs and other compact networks have been proposed for UAV classification from micro-Doppler measurements [13,14], while specialized relation, residual, and fusion networks have been used to model radar target structure [15,16]. These studies provide examples of radar classifiers designed for computational efficiency or radar-specific feature modeling.

The present work employs a compact scratch-trained depthwise CNN as the window-level classifier within the radar classification pipeline. Its role is to provide a controlled low-parameter model for evaluating the proposed observation interface and the subsequent decision-stage temporal aggregation, rather than to introduce a new radar backbone architecture.

### 2.4. Temporal Evidence Aggregation in Radar Classification

Micro-Doppler observations are temporally ordered, and discriminative information can vary across neighboring short-time windows. Prior work has exploited temporal structure through temporal convolution for radar micro-Doppler gait recognition and automotive-radar target classification [17,18], while millimeter-wave radar gesture sensing further demonstrates the value of modeling structured motion over time [31]. In UAV classification, feature- and decision-level fusion strategies have also been explored to combine complementary radar evidence [19,20].

In this study, temporal evidence is incorporated at the decision level rather than through a separately trained sequence model. The trained single-window classifier is kept fixed, and neighboring window outputs are combined only as offline post-processing. This design distinguishes the evaluated fusion stage from causal sequential inference and from trainable temporal modeling.

### 2.5. Recording-Disjoint Evaluation and Dataset Leakage Risk

Windowed sensing datasets require a clear definition of the evaluation unit. When many overlapping windows originate from one recording, random allocation of individual windows can place closely related observations in both training and test sets. General machine learning methodology identifies non-independent samples across train and test sets as a source of leakage and reduced reproducibility [21]. For the held-out-recording evaluation considered here, source-recording identity is used as the grouping unit rather than individual windows.

Recording-disjoint splitting removes direct source-recording overlap but does not constitute controlled-condition or cross-domain validation. Acquisition session, nominal range, viewing geometry, radar configuration, or other dataset-specific conditions may still be correlated with the held-out recordings. The protocol therefore defines a within-dataset held-out-recording evaluation setting rather than establishing generalization across unmatched ranges, sessions, sites, or radar systems.

### 2.6. Positioning of This Study

Prior work has advanced observation design, compact classifiers, temporal fusion, and recording-disjoint evaluation as separate directions, but no single study has coupled them under one protocol. This study fills that gap with a deterministically constructed three-channel observation interface, a scratch-trained compact depthwise CNN, offline temporal logit fusion, and recording-disjoint evaluation. Each component is selected under the constraints imposed by the others.

As summarized in Table 1, the two decisions at the center of this study are observation-interface design and classifier design. This study integrates the two under a single controlled protocol, and the integration is technically coupled rather than a loose combination. The observation interface is deterministic by construction, which allows the classifier to be trained from scratch without large-scale pretraining. The resulting compact depthwise CNN has a restricted parameter budget, which motivates temporal logit fusion as a post-processing step that compensates for single-window discriminative limits. In addition, the fusion operates within each recording, which makes the recording-disjoint protocol necessary to verify that the observed gain reflects genuine cross-recording generalization rather than within-recording information leakage. The present work is positioned as a controlled empirical evaluation rather than an end-to-end detector, a new temporal network, or a broad representation survey. It first compares the proposed deterministic three-channel observation interface with a repeated-channel reference, and then evaluates offline temporal logit fusion as a post-processing step applied to the same compact single-window classifier. This positioning separates observation construction, window-level classification, and decision-stage temporal aggregation while keeping the evaluation aligned with a held-out-recording classification question.

## 3. Materials and Methods

### 3.1. Task Definition and System Boundary

This study addresses closed-set UAV classification after a relevant FMCW radar target return has been localized in range. The evaluated classification stage takes a target-range-bin echo segment as input and predicts one of six known UAV types. Target detection, target-presence estimation, automatic range localization, multi-target association, and range-bin tracking are treated as upstream stages and are not evaluated in this study.

For each window indexed by *t*, the processing chain is written as

(1) $$E_{t}\to S_{\mathrm{norm},t}\to X_{t}\to F_{t}\to z_{t}\to {\bar{z}}_{t}\to {\hat{y}}_{t},$$

where $E_{t}$ denotes the range-localized target echo segment, $S_{\mathrm{norm},t}$ is the normalized micro-Doppler magnitude map, $X_{t}$ is the three-channel classifier observation, and $F_{t}$ denotes the depthwise CNN feature maps. The vector $z_{t}$ is the window-level logit vector produced by the classifier. The vector ${\bar{z}}_{t}$ denotes the decision-stage logit vector after optional temporal fusion; for single-window inference, ${\bar{z}}_{t}=z_{t}$. Since the temporal fusion evaluated in this study uses centered neighborhoods, fused configurations with K>1 are offline post-processing operations rather than causal sequential inference. Figure 1 summarizes the post-localization classification pipeline from the range-localized echo segment to the class decision; the remainder of this section details each stage.

### 3.2. Dataset and Recording-Disjoint Evaluation Protocol

Experiments are conducted on LSS-FMCWR-1.0, a public FMCW radar dataset for low-slow-small targets [24]. The dataset contains FMCW radar echo recordings from six UAV types collected under different radar modulation settings and acquisition conditions. The dataset was collected using a K-band FMCW radar system and contains raw complex I/Q echo recordings stored in MATLAB R2021b MAT-files, with each file containing the multi-channel radar echo data organized by slow-time chirps. In this study, six UAV classes are considered, DJI Mavic 2, DJI Phantom, DJI M350, DJI Inspire 2, DJI M600, and WL F949s, encoded as class IDs 0–5, respectively. Table 2 summarizes the acquisition details of the selected recordings used in this study. All selected recordings share the same modulation period (0.3 ms), bandwidth (100 MHz), and radar band (K-band), while nominal target ranges vary between 8 m and 12 m across classes. The evaluated task is formulated as a six-class closed-set classification problem, where each input window is assigned to one predefined UAV class.

The complete LSS-FMCWR-1.0 dataset contains 114 FMCW radar data recordings. The dataset naming convention encodes the UAV category, modulation period, bandwidth, target range, radar band, and recording index, enabling the selection of subsets with consistent acquisition configurations. For example, 01-2023.5.8-0.3-100-9-K (1).mat denotes class 01, collected on 8 May 2023, with 0.3  ms modulation period, 100 MHz bandwidth, 9 m nominal range, K-band, trial 1. The dataset is publicly available at https://radars.ac.cn/web/data/getData?newsColumnId=62117dab-1688-4a5e-834d-646b09724520 (accessed on 15 July 2025). To ensure a balanced evaluation across UAV classes, this study selects a subset acquired under the same radar parameter configuration (0.3 ms modulation period, 100 MHz bandwidth, K-band, with nominal target ranges of 8–12 m), retaining three source MAT recordings for each of the six classes. This results in 18 selected source recordings and provides an identical number of recording instances for every class.

The source MAT recording is treated as the fundamental grouping unit for data splitting. Each selected recording is divided into temporally ordered windows, and all derived windows inherit the UAV class label and source-recording identity of their originating MAT file. Since adjacent windows from the same recording exhibit strong temporal correlation, splitting at the window level would introduce potential information leakage. Therefore, all windows generated from one source recording are assigned exclusively to either the training or testing partition.

A three-fold recording-disjoint leave-one-recording-out protocol is adopted over the selected recordings. For each UAV class, three source recordings are available. In Fold 1, the first recording is held out for testing while the remaining two recordings are used for training; Fold 2 and Fold 3 repeat the procedure by holding out the second and third recordings, respectively. Consequently, each selected source recording serves as the test recording exactly once across the three folds, and no source recording is shared between training and testing within any fold. The resulting split structure is illustrated in Figure 2.

Within each fold, 300 training windows and 150 test windows are sampled for each class, resulting in 1800 training windows and 900 test windows in total. Test windows are sampled exclusively from the held-out source recording of each class. Training windows are sampled from the union of candidate windows generated from the two training recordings of the same class, without enforcing equal contributions from individual recordings. No validation subset is used. The data-sampling seed is fixed to 123. For each recording-disjoint fold, three model initialization seeds (0, 1, and 2) are evaluated, producing nine fold–seed outcomes for descriptive reporting.

This protocol eliminates direct source-recording overlap between training and testing and provides a controlled evaluation of UAV classification performance under matched radar acquisition conditions. However, the protocol does not remove all possible variations associated with acquisition session, target range, viewing geometry, or other measurement factors present in the original dataset. Therefore, the reported results characterize generalization to held-out source recordings within the evaluated subset rather than unrestricted generalization across unmatched sensing conditions, radar configurations, or acquisition environments.

### 3.3. Range-Localized Echo Preparation

The LSS-FMCWR-1.0 dataset provides raw FMCW radar echo recordings together with a signal-processing convention for range-domain target extraction [24]. This study uses the complex I/Q measurements stored in echoes.channelA. For each selected slow-time window, range-domain processing is applied to obtain the complex echo sequence associated with the target range bin.

The target range is not estimated by the classifier. Instead, the nominal target distance encoded in the source MAT filename is used to select the nearest available range bin. For a nominal target distance $d_{t}^{\mathrm{nom}}$, the selected range bin is determined by

(2) $$k_{t}^{*}=\arg\min_{k}\left|R_{k}-d_{t}^{\mathrm{nom}}\right|,$$

where $R_{k}$ denotes the range axis generated by the range-processing procedure. The corresponding complex slow-time sequence is then used for micro-Doppler observation formation.

Accordingly, the reported performance characterizes UAV classification given a metadata-based range-localized target return. Automatic target detection, target-range estimation, and range-bin association remain outside the evaluated system boundary.

### 3.4. Micro-Doppler Observation Formation

For each range-localized target echo segment $E_{t}$, a micro-Doppler observation is generated by short-time Fourier transformation, logarithmic amplitude compression, and per-window normalization. A polynomial detrend of degree 5 is applied to the target-bin slow-time sequence before the STFT to remove slowly varying baseline drift. The STFT is implemented using scipy.signal.stft with a Hamming window of 128 samples, an overlap of 127 samples, and an FFT length of 1024. The computation uses return_onesided=False, boundary=None, and padded=False, retaining the two-sided Doppler-frequency representation without Doppler-bin cropping.

Let $Z_{t}$ denote the complex STFT output. The magnitude representation is

(3) $$A_{t}=\left|Z_{t}\right|.$$

The amplitude map is converted to a logarithmic representation using per-window maximum normalization:

(4) $$L_{t}=\log\left(\frac{A_{t}}{\max_{p,q}A_{t,pq}+\epsilon_{\log}}+\epsilon_{\log}\right),$$

where *p* and *q* index Doppler-frequency and slow-time dimensions, respectively, and $\epsilon_{\log}=10^{-8}$.

The logarithmic spectrogram is then normalized by per-window min–max scaling:

(5) $$S_{\mathrm{norm},t}=\frac{L_{t}-\min_{p,q}L_{t,pq}}{\max_{p,q}L_{t,pq}-\min_{p,q}L_{t,pq}+10^{-8}}.$$

The normalized spectrogram is resized to 224×224 using bilinear interpolation. Rows correspond to Doppler-frequency bins and columns correspond to slow-time frames.

The deterministic three-channel classifier input is constructed from $S_{\mathrm{norm},t}$ as described in Section 3.5. The observation-formation configuration is fixed for all repeated-channel, proposed single-window, and temporal-fusion experiments. Table 3 summarizes the STFT and normalization parameters.

### 3.5. Deterministic Three-Channel Micro-Doppler Representation

The classifier receives a deterministic three-channel representation derived from the same normalized micro-Doppler spectrogram. The three channels encode the normalized magnitude map, a signed row-centered residual, and a signed temporal-gradient map. These channels are fixed transformations of a single radar observation rather than independent sensing bands or RGB color components.

Let

(6) $$S_{\mathrm{norm},t}\in {\left[0,1\right]}^{H\times W},\;H=W=224,$$

denote the normalized micro-Doppler spectrogram obtained in Section 3.4. The first classifier input channel is the normalized magnitude map mapped to the signed interval [−1,1]:

(7) $$C_{1,t}=2S_{\mathrm{norm},t}-1.$$

The second channel is a signed row-centered residual. For Doppler-frequency row *i*, the median response over slow time is

(8) $$m_{t,i}={\mathrm{median}}_{j=1,\ldots ,W}S_{\mathrm{norm},t}\left(i,j\right).$$

The residual is

(9) $$R_{t}\left(i,j\right)=S_{\mathrm{norm},t}\left(i,j\right)-m_{t,i},$$

and the row-centered channel is obtained by sample-wise maximum-absolute normalization:

(10) $$C_{2,t}\left(i,j\right)=\frac{R_{t}\left(i,j\right)}{\max_{p,q}\left|R_{t}\left(p,q\right)\right|+\epsilon_{\mathrm{ch}}},\;\epsilon_{\mathrm{ch}}=10^{-6}.$$

Centering each Doppler row by its median is intended to suppress static or slowly varying row-level offsets, such as those produced by strong body or stationary-clutter returns, so that the row-wise relative fluctuations induced by blade modulation become more prominent; the transformation is deterministic and row-wise and introduces no learnable parameters.

The third channel captures local variation along slow time. Let $D_{t}\left(i,j\right)$ denote the finite difference of $S_{\mathrm{norm},t}$ along the slow-time dimension:

(11) $$D_{t}\left(i,j\right)=\left\{\begin{array}{cc} S_{\mathrm{norm},t}\left(i,2\right)-S_{\mathrm{norm},t}\left(i,1\right), & j=1,\\ \frac{1}{2}\left[S_{\mathrm{norm},t}\left(i,j+1\right)-S_{\mathrm{norm},t}\left(i,j-1\right)\right], & 1<j<W,\\ S_{\mathrm{norm},t}\left(i,W\right)-S_{\mathrm{norm},t}\left(i,W-1\right), & j=W. \end{array}\right.$$

The signed temporal-gradient channel is

(12) $$C_{3,t}\left(i,j\right)=\frac{D_{t}\left(i,j\right)}{\max_{p,q}\left|D_{t}\left(p,q\right)\right|+\epsilon_{\mathrm{ch}}}.$$

The first-order difference along slow time is intended to make the channel more sensitive to the temporal structure of periodic blade flashes and their harmonics, which modulate the envelope at frame-to-frame rates; because the sign is retained, the direction of approaching–receding Doppler variation is not folded away. This design rationale is consistent with the per-channel ablation of Section 4.1 but is not separately verified by a controlled experiment in this study, and the ablation is not claimed to prove the underlying physical mechanism. No absolute-value transformation or non-negative remapping is applied to $C_{2,t}$ or $C_{3,t}$, so the signs of the residual and temporal variation are retained.

The resulting classifier input is

(13) $$X_{t}=\left(C_{1,t},C_{2,t},C_{3,t}\right)\in R^{3\times H\times W}.$$

For the component ablation, a repeated-channel reference is also evaluated. This reference repeats the same magnitude-derived input plane across the three classifier channels after the same normalization and standardization pipeline, while keeping the input size, depthwise CNN architecture, training schedule, recording-disjoint folds, and model seeds unchanged. It is used to contextualize the proposed observation interface; all temporal-neighborhood analyses use the proposed three-channel representation in Equation (13).

Figure 3 illustrates the three deterministic views derived from the same normalized micro-Doppler observation.

### 3.6. Compact Depthwise CNN Backbone

A compact depthwise CNN is employed to extract discriminative features from the deterministic three-channel micro-Doppler representation. The network is trained from random initialization and consists of a convolutional stem, four depthwise-separable convolutional blocks, and a single classification head. Depthwise-separable convolutions are adopted to reduce model complexity while maintaining effective feature extraction over the Doppler-frequency and slow-time dimensions.

Given the three-channel observation

(14) $$X_{t}\in R^{3\times 224\times 224},$$

the convolutional stem first applies a 3×3 convolution with 24 output channels, stride 2, and padding 1, followed by batch normalization and ReLU activation:

(15) $$H_{t}^{\left(0\right)}=\mathrm{ReLU}\left(\mathrm{BN}\left({\mathrm{Conv}}_{3\times 3}\left(X_{t}\right)\right)\right).$$

This operation reduces the spatial resolution from 224×224 to 112×112.

The subsequent feature extractor consists of four depthwise-separable convolutional blocks with channel transitions

(16) 24→48,48→96,96→160,160→160.

Each block performs spatial filtering and channel mixing through a depthwise convolution followed by a pointwise convolution. Specifically, each block follows the sequence

(17) $${\mathrm{DWConv}}_{3\times 3}\to \mathrm{BN}\to \mathrm{ReLU}\to {\mathrm{PWConv}}_{1\times 1}\to \mathrm{BN}\to \mathrm{ReLU}.$$

The depthwise convolution operates independently on each input channel, while the pointwise convolution performs cross-channel feature integration. The first two depthwise-separable blocks use stride 2 for spatial downsampling, whereas the final two blocks use stride 1 to preserve the spatial resolution.

The resulting feature tensor is therefore

(18) $$F_{t}=B\left(X_{t}\right)\in R^{160\times 28\times 28},$$

where B(·) denotes the depthwise CNN feature extractor. The spatial resolution progression through the backbone is

(19) 112×112→56×56→28×28→28×28→28×28.

Adaptive global average pooling is then applied to obtain a compact feature vector:

(20) $$h_{t}=\mathrm{GAP}\left(F_{t}\right)\in R^{160}.$$

During training, dropout with probability 0.1 is applied before the final classifier. The window-level class logits are computed as

(21) $$z_{t}=W_{c}\mathrm{Dropout}\left(h_{t}\right)+b_{c}\in R^{6},$$

where $W_{c}$ and $b_{c}$ denote the parameters of the final linear classifier. The resulting $z_{t}$ represents the six-class prediction logits for the corresponding radar observation window and is subsequently used in the decision stage.

The complete network contains 52,918 trainable parameters. Convolutional layers in the stem and depthwise-separable blocks use no bias terms because they are followed by batch normalization, whose affine parameters remain trainable. The final linear classifier includes a bias term. Table 4 summarizes the complete architecture.

### 3.7. Training Protocol

Except for the repeated-channel input used only in the component ablation, all reported classifier runs use the deterministic three-channel representation described in Section 3.5 and the compact depthwise CNN described in Section 3.6. All models are trained from random initialization without pretrained weights or initialization from previous experimental runs. Stochastic data augmentation is not applied during training.

Each model is trained for 20 epochs using the AdamW optimizer [32] with an initial learning rate of $3\times 10^{-4}$, weight decay of $5\times 10^{-4}$, batch size of 16, and gradient clipping with a maximum norm of 1.0. The AdamW coefficients are set to $\left(\beta_{1},\beta_{2}\right)=\left(0.9,0.999\right)$ with $\epsilon =10^{-8}$. The training objective is cross-entropy loss with label smoothing of 0.05. The classifier dropout probability is fixed at 0.1. A ReduceLROnPlateau scheduler monitors the training loss and reduces the learning rate by a factor of 0.5 after four consecutive epochs without improvement, with a minimum learning rate of $10^{-6}$.

No validation subset is introduced for model selection, and neither early stopping nor best-validation checkpoint selection is performed. Therefore, the final model checkpoint after epoch 20 is used for evaluation. The recording-disjoint data partition is generated using a fixed split seed of 123, while model seeds 0, 1, and 2 are independently applied to each of the three folds.

The fixed-epoch design is supported by the observed convergence behavior of the nine fold–seed runs. Figure 4 shows the per-epoch training loss and training accuracy of all nine runs of the main experiment: the training loss decreases sharply over the first epochs and then stabilizes near the label-smoothing floor of the cross-entropy objective, while the mean training accuracy exceeds 99% from epoch 4 onward and all nine runs reach 100% training accuracy by the final epoch (final-epoch training loss 0.258±0.001 and final-epoch training accuracy 100.00±0.00%, mean ± std across the nine runs). The small gap between the final training accuracy and the held-out-recording test accuracy (98.36±0.89%) indicates that the 20-epoch budget converges without pronounced overfitting under the evaluated protocol. Fixing the epoch budget also keeps the training procedure identical across all compared configurations, so that the representation, ablation, and temporal-fusion comparisons are not confounded by variable early-stopping or validation-based model-selection criteria. Because this protocol uses no validation split, the training loss is the only monitoring signal available to the scheduler, so the schedule forms part of the fixed-epoch budget rather than a model-selection criterion. No best-epoch selection on any held-out or test metric is performed: for all nine fold–seed runs the final-epoch checkpoint (epoch 20) is the checkpoint used for every reported evaluation, so the reported results cannot benefit from validation-informed or test-informed model selection. Validation-based training schedules remain a direction for future work (Section 5.6).

The model seed controls stochastic factors associated with training, including parameter initialization, sample ordering, dropout, and other random operations during optimization. Deterministic execution settings are enabled where supported to reduce variability between repeated runs. However, exact bitwise reproducibility across different hardware, CUDA libraries, and software environments is not assumed.

All training settings are kept identical across the nine fold–seed runs. The model size is fixed at 52,918 trainable parameters, as reported in Table 4; computational latency and inference time are not evaluated in this study. The implementation environment uses Python 3.10.16, PyTorch 2.5.1, NumPy 1.26.4, SciPy 1.15.3, and scikit-learn 1.7.0.

### 3.8. Offline Centered Within-Recording Temporal Logit Fusion

The trained depthwise CNN produces a logit vector independently for each radar observation window. In the preceding formulation, the recording index is omitted because each window is processed independently. To make the recording boundaries explicit for temporal fusion, the window-level logits are here indexed as

(22) $$z_{g,t}\in R^{C},\;C=6,$$

where *g* denotes the source recording and *t* denotes the temporally ordered window within that recording. Thus, $z_{g,t}$ corresponds to the window-level logit vector $z_{t}$ defined in Equation (21), with the additional recording index introduced specifically for the temporal-fusion stage. Windows are ordered according to their start positions within each recording, and temporal fusion is performed independently for each recording.

For an odd neighborhood length K=2r+1, the centered temporal neighborhood associated with window *t* is defined as

(23) $$\Omega_{g,t}^{\left(K\right)}=\left\{\tau :\;1\leq \tau \leq T_{g},\;\left|\tau -t\right|\leq r\right\},$$

where $T_{g}$ denotes the number of evaluated windows in recording *g*. By construction, $\Omega_{g,t}^{\left(K\right)}$ contains only windows from the same source recording and therefore never crosses a recording boundary.

The temporally fused logit vector is obtained by averaging the window-level logits within the centered neighborhood:

(24) $${\bar{z}}_{g,t}^{\left(K\right)}=\frac{1}{|\Omega_{g,t}^{\left(K\right)}|}\sum_{\tau \in \Omega_{g,t}^{\left(K\right)}}z_{g,\tau }.$$

The corresponding class prediction is

(25) $${\hat{y}}_{g,t}^{\left(K\right)}=\underset{1\leq n\leq C}{arg max}{\left[{\bar{z}}_{g,t}^{\left(K\right)}\right]}_{n}.$$

At the beginning and end of a recording, the centered neighborhood is truncated to the available windows. No temporal padding is introduced, and logits from different recordings are never combined. The neighborhood lengths

(26) K∈{1,3,5,7,9}

are evaluated to examine the effect of temporal context. The K=1 condition corresponds exactly to single-window inference without temporal aggregation. The K=3 condition is used as the reported short-neighborhood fusion configuration, whereas K∈{5,7,9} is evaluated only to characterize sensitivity to the temporal neighborhood length. For the reported K=3 configuration, the superscript (K) is omitted when no ambiguity arises, yielding the notation ${\bar{z}}_{g,t}$ and ${\hat{y}}_{g,t}$.

Because K>1 defines a centered neighborhood, the fused prediction may depend on both preceding and subsequent windows from the same recording. The proposed temporal logit fusion is therefore an offline, non-causal post-processing procedure rather than an online inference mechanism. It is applied only after all required single-window logits have been produced, introduces no additional trainable parameters, and does not modify the training procedure. Fusion is performed directly in logit space, without applying softmax before averaging. Ground-truth class labels are not used to define the temporal neighborhoods or to compute the fused logits.

For online deployment, the centered neighborhood can be replaced by a causal trailing variant that aggregates only the current and two preceding windows (τ∈{t−2,t−1,t}) from the same recording. This removes any dependence on future windows, so no lookahead is required, at the cost of shifting the effective aggregation center one window earlier than in the centered configuration. Because the trailing neighborhood is one-sided, it provides less symmetric temporal evidence than the centered window, so its accuracy is expected to be slightly lower; this study does not quantify the causal variant and makes no numerical performance claim for it. The causal form is intended for radar scenarios that require real-time, frame-by-frame decisions rather than offline analysis.

### 3.9. Metrics and Statistical Reporting

Classification performance is evaluated primarily using macro-F1, with accuracy reported as a secondary aggregate metric. Class-wise F1 scores and pooled confusion matrices are additionally used to examine class-specific performance and error patterns. For class *c*, precision, recall, and F1 are defined as

(27) $$P_{c}=\frac{TP_{c}}{TP_{c}+FP_{c}},\;R_{c}=\frac{TP_{c}}{TP_{c}+FN_{c}},$$

and

(28) $$F1_{c}=\frac{2P_{c}R_{c}}{P_{c}+R_{c}}.$$

If a denominator is zero, the corresponding precision, recall, or F1 value is defined as 0. For the C=6 UAV classes, macro-F1 is computed as

(29) $$\mathrm{MacroF}1=\frac{1}{C}\sum_{c=1}^{C}F1_{c},$$

and classification accuracy is computed as

(30) $$\mathrm{Accuracy}=\frac{N_{\mathrm{correct}}}{N_{\mathrm{total}}}.$$

Each fold–seed run contains 150 test windows for each of the six UAV classes. Therefore, weighted-F1 is numerically identical to macro-F1 under the present evaluation protocol and is not reported as a separate performance measure.

Unless otherwise stated, aggregate performance is summarized as the mean ± sample standard deviation across the nine fold–seed runs. For run-level measurements $x_{1},\ldots ,x_{n}$, where n=9, the sample mean and sample standard deviation are

(31) $$\bar{x}=\frac{1}{n}\sum_{i=1}^{n}x_{i},$$

and

(32) $$s=\sqrt{\frac{1}{n-1}\sum_{i=1}^{n}{\left(x_{i}-\bar{x}\right)}^{2}}.$$

The nine outcomes arise from three model seeds evaluated within each of the three recording-disjoint folds. They are treated as descriptive repeated outcomes rather than as nine independent datasets. The reported sample standard deviation therefore describes variability across fold–seed runs and is not interpreted as a confidence interval. No inferential hypothesis testing is performed.

Temporal-neighborhood sensitivity is evaluated using matched run-level comparisons relative to the K=1 single-window condition. For each comparison, the held-out recording fold, trained classifier, and model seed are fixed, while only the temporal-fusion length *K* is varied. This design isolates the descriptive effect of applying different centered within-recording temporal neighborhoods to the same set of single-window logits.

The component ablation is summarized over the same recording-disjoint fold–seed grid using three matched configurations: repeated-channel single-window input, proposed three-channel K=1 single-window input, and proposed three-channel input with the reported K=3 offline fusion. This comparison is descriptive and keeps the classifier architecture and training protocol fixed except for the input representation or final fusion stage being evaluated. In addition, a per-channel ablation examines seven input-channel configurations obtained by zeroing individual channels of the proposed three-channel input while keeping the stem, architecture, and training protocol unchanged; this grid is likewise summarized over the same nine fold–seed runs.

For class-specific analysis, class-wise F1 scores and the pooled confusion matrix are reported for the K=1 single-window reference. Class-wise F1 is computed independently for each fold–seed run and summarized descriptively. The pooled confusion matrix is obtained by summing the nine K=1 confusion-count matrices element-wise and then normalizing each row so that it represents the empirical distribution of predicted classes for each true class across the complete set of fold–seed evaluations.

## 4. Results

All results are reported under the recording-disjoint evaluation protocol described in Section 3.2, the training protocol described in Section 3.7, and the statistical reporting procedure defined in Section 3.9. Unless otherwise stated, aggregate values are summarized as mean ± sample standard deviation across the nine fold–seed runs.

### 4.1. Component Contribution Ablation

A component-level ablation was used to place the proposed observation interface and the final temporal-fusion stage in the same descriptive comparison. Using the same compact depthwise CNN architecture and recording-disjoint fold–seed protocol, the repeated-channel K=1 reference achieved a mean macro-F1 of 97.67%. The proposed deterministic three-channel representation achieved a higher mean macro-F1 of 98.35% under K=1 single-window inference. Applying the reported K=3 offline temporal logit fusion to the proposed representation further raised mean macro-F1 to 98.60%. Thus, the ablation shows a descriptive gain from replacing simple channel repetition with the proposed observation interface, followed by a smaller additional difference from short-neighborhood logit fusion. Figure 5 summarizes this component-level comparison.

The leave-one-channel-out configurations extend this comparison to the individual channels of the observation interface. Channel ablations are realized by scaling the corresponding input channel to exactly zero while keeping the three-channel stem, the depthwise CNN architecture, and all training hyper-parameters unchanged; no reduced-channel network is re-instantiated. All seven configurations therefore have identical parameter counts (52,918), and any performance difference is attributable solely to the information content of the retained channels. Table 5 summarizes these seven configurations.

The full configuration achieves the highest mean macro-F1 across the nine fold–seed runs (98.35±0.90%), and no reduced configuration reaches its mean. Removing any single channel causes only a small decrease in mean macro-F1 (0.28–0.81 percentage points for the three two-channel configurations). The channels nevertheless behave asymmetrically: the temporal-gradient channel C3 alone retains 98.00±1.12%, and the magnitude channel C1 alone 97.87±0.60%, whereas the row-centered residual channel C2 alone drops to 93.98±2.64%, the only large and direction-consistent difference in the comparison. These differences are small relative to the fold–seed variability and are reported descriptively rather than as significance claims.

### 4.2. Single-Window and Reported Fused Performance

For the proposed three-channel configuration, the K=1 single-window reference achieved 98.36±0.89% accuracy and 98.35±0.90% macro-F1. Applying offline centered within-recording temporal logit fusion with the reported neighborhood length K=3 yielded 98.62±1.02% accuracy and 98.60±1.04% macro-F1.

Within matched fold–seed pairs, the change in macro-F1 from K=1 to K=3 was +0.26±0.33 percentage points. Of the nine paired comparisons, six showed an increase, one was unchanged, and two showed a decrease. Temporal logit fusion introduces no additional trainable parameters and operates only on the single-window logits produced by the trained classifier. The K=3 setting is treated as the reported short-neighborhood fused configuration rather than as an empirically selected optimum. Table 6 summarizes the two configurations.

Figure 6 shows the fold–seed variation in the proposed K=1 single-window results underlying the aggregate reference performance.

### 4.3. Temporal-Neighborhood Sensitivity

To characterize sensitivity to the temporal neighborhood length, the same single-window logits were evaluated using K∈{1,3,5,7,9}. Mean macro-F1 was 98.35±0.90% at K=1, 98.60±1.04% at K=3, 98.69±1.08% at K=5, 98.75±1.08% at K=7, and 98.77±1.05% at K=9. Relative to the matched K=1 reference, the paired changes in macro-F1 were +0.26±0.33, +0.35±0.36, +0.41±0.44, and +0.42±0.46 percentage points for K=3, 5, 7, and 9, respectively.

The corresponding positive/unchanged/negative paired-change counts were 6/1/2 for K=3, 8/0/1 for K=5, 7/2/0 for K=7, and 8/1/0 for K=9. Although the mean macro-F1 values were modestly higher for longer neighborhoods, the incremental change became small at the longer settings. For example, the mean difference between K=7 and K=9 was only 0.01 percentage points. The longer-neighborhood results are therefore used only to characterize sensitivity to temporal context and are not interpreted as alternative primary configurations or as evidence that K=9 is an optimal neighborhood length. Table 7 and Figure 7 summarize these results.

### 4.4. Class-Wise Error Analysis

Class-specific performance and confusion patterns are examined using the K=1 single-window reference. The mean class-wise F1 was highest for DJI M350 at 99.81±0.30% followed by DJI Inspire 2 at 99.55±0.65%, DJI Mavic 2 at 99.08±1.67%, WL F949s at 98.70±2.43%, DJI M600 at 97.88±2.35%, and DJI Phantom at 95.05±3.05%.The mean class-wise F1 was highest for DJI Inspire 2 at 100.00%, followed by WL F949s at 99.82%, DJI Mavic 2 at 98.04%, DJI M350 at 97.57%, and DJI Phantom and DJI M600 at 96.39% each. Class-wise F1 values are reported as per-run means across the nine fold–seed runs, and the matrix cells as run-summed confusion counts normalized by row.

The pooled K=1 confusion matrix shows that the largest row-normalized off-diagonal entries involved DJI Phantom predicted as DJI M600 at 4.07% and DJI Phantom predicted as WL F949s at 2.74%, followed by DJI Mavic 2 predicted as DJI Phantom at 1.78%. The remaining off-diagonal entries were below 1%, including DJI Phantom predicted as DJI Inspire 2 at 0.52%, DJI Inspire 2 predicted as DJI Phantom at 0.37%, DJI M350 predicted as DJI M600 at 0.30%, and DJI M600 predicted as DJI M350 at 0.07%. Because each fold-specific test set is evaluated independently by three model seeds, the pooled percentages summarize repeated model decisions rather than independent recording counts. This class-specific analysis is restricted to the K=1 single-window reference and does not represent the temporally fused K=3 predictions. Figure 8 summarizes the class-wise F1 scores and the pooled confusion pattern.

### 4.5. Expanded-Subset Evaluation

The main results are obtained from a balanced 18-recording subset selected from LSS-FMCWR-1.0 under a single radar configuration (0.3 ms modulation period, 100 MHz bandwidth, K-band). To provide initial evidence on sensitivity to radar acquisition parameters, the same depthwise CNN architecture and three-channel observation interface were evaluated on an expanded subset drawn from recordings with varied modulation periods, frequency bands, and nominal ranges.

The expanded subset retains the six-class structure and the recording-disjoint protocol of the main experiment: three source MAT recordings per class (18 recordings in total) are selected by filename, and in each fold one recording per class is held out for testing while the remaining two provide training windows. The classifier is trained from scratch on each fold under the same schedule as the main experiment. Table 8 lists the per-class radar parameters parsed from the selected source filenames. Four classes (DJI Mavic 2, DJI M350, DJI Inspire 2, and DJI M600) use recordings with a 1.024 ms modulation period and L-band radar; DJI Phantom uses 1.024 ms with K-band, and WL F949s uses 0.3 ms with L-band. Nominal target ranges span 6 m to 13 m. Relative to the main configuration (0.3 ms, K-band), the expanded subset changes the modulation period in five of six classes and the frequency band in five of six classes, with four classes changing both.

Table 9 summarizes the per-fold results. The mean accuracy was 96.91±3.14% and the mean macro-F1 was 96.82±3.29% across the nine fold–seed runs. Compared with the main experiment (98.36±0.89% accuracy, 98.35±0.90% macro-F1), the expanded subset showed a decrease of about 1.5 percentage points in mean performance and higher fold-to-fold variability, concentrated in Fold 1.

The Fold 1 decrease is driven almost entirely by a single confusion pattern. In the pooled Fold 1 confusion matrix, 181 of the 450 DJI Mavic 2 test windows (40.2%) were predicted as DJI Phantom (46, 71, and 64 windows for model seeds 0, 1, and 2, respectively), while the remaining off-diagonal entries were minor (six DJI M350 windows predicted as DJI Phantom and one DJI Phantom window predicted as WL F949s). This confusion did not dominate the other folds: all 450 DJI Mavic 2 test windows of Fold 2 were classified correctly, and in Fold 3 only 42 of 450 DJI Mavic 2 windows (9.3%) were predicted as DJI M350. The Fold 1 result is therefore specific to the held-out recording (1.024 ms, L-band, 9 m) rather than a uniform degradation across all expanded acquisition conditions.

## 5. Discussion

### 5.1. Main Findings and Role of the Compact Classifier

The results show that high closed-set UAV classification performance can be obtained with a compact scratch-trained depthwise CNN under the evaluated recording-disjoint, post-localization protocol. The classifier serves as a controlled window-level model for comparing observation interfaces and decision-stage temporal fusion, rather than as an exhaustive study of radar backbone architectures. Its parameter count documents model scale and supports reproducibility, while latency and deployment efficiency remain implementation-specific topics for future evaluation.

The component ablation supports the use of the proposed deterministic three-channel micro-Doppler observation interface. Under the same classifier architecture, training schedule, fold–seed grid, and recording-disjoint protocol, the proposed representation achieved higher macro-F1 than the repeated-channel reference. This suggests that adding signed row-centered residual and temporal-gradient information provides useful structure beyond simple replication of the magnitude-derived input plane. The per-channel ablation shows that the three channels are not redundant copies of the same information: removing the magnitude channel costs more than removing either signed channel, the temporal-gradient channel alone nearly matches the full configuration, and the row-centered residual channel alone collapses. The full configuration nevertheless remains the highest-mean configuration, and it exceeds each of the three two-channel configurations in the majority of paired fold–seed runs (7 of 9 against C1 + C2, 6 of 9 against C1 + C3, and 7 of 9 against C2 + C3), although the mean paired gains of 0.30, 0.28, and 0.81 percentage points are each smaller than the corresponding paired standard deviations of 0.45, 0.91, and 1.47. The results therefore support the three-channel design as better on average rather than as a large-margin or statistically significant improvement.

### 5.2. Value and Boundary of Recording-Disjoint Evaluation

The recording-disjoint protocol is an important part of the evaluation design. Temporally adjacent windows extracted from the same radar recording share source acquisition conditions and overlapping slow-time content. If such windows were randomly distributed between training and testing, the test partition could partially reflect source-recording similarity rather than held-out-recording performance. Grouping by source MAT recording removes this direct train–test overlap within each fold and better matches the stated held-out-recording classification question.

At the same time, recording-disjoint evaluation remains a within-dataset assessment. The held-out recordings are still drawn from the same selected subset and may share acquisition-related factors such as nominal range, viewing geometry, radar configuration, background, or session conditions. The reported results therefore characterize post-localization classification within the evaluated dataset protocol. Generalization across radar systems, sites, ranges, sessions, or open-environment conditions would require additional controlled evaluation.

### 5.3. Sensitivity to Radar Acquisition Parameters

The expanded-subset experiment provides initial evidence on how the evaluated pipeline behaves when the radar acquisition parameters differ across the recordings used. The observed decrease of about 1.5 percentage points in mean performance and the higher fold-to-fold variability are concentrated in Fold 1, whose held-out DJI Mavic 2 recording (1.024 ms, L-band, 9 m) was frequently confused with DJI Phantom. The other folds, whose held-out recordings covered the same range of parameter variation, classified at a level close to the main experiment. This pattern indicates that the observed difference reflects recording-specific behavior within the small expanded subset rather than a systematic performance limit of the architecture under parameter variation.

These results are therefore best read as an initial probe of configuration sensitivity. The experiment shows that the same architecture and protocol can be applied to recordings with different modulation periods, frequency bands, and ranges, and that most held-out recordings under the varied parameters were classified at a level comparable to the main subset. At the same time, the single-confusion-dominated Fold 1 result illustrates the boundary of the evidence: the expanded subset contains only three recordings per class, so the per-fold estimates are sensitive to individual recordings, and the results do not establish quantitative generalization guarantees across radar configurations.

Two directions follow for future work. First, multi-configuration training, in which the training windows are drawn from recordings with different radar parameters, could improve robustness to parameter variation and deserves a dedicated evaluation. Second, benchmark datasets that systematically vary radar parameters would enable more rigorous assessment of how classification performance depends on individual acquisition factors.

### 5.4. Offline Temporal Evidence Aggregation

The temporal-fusion results indicate that decision-stage averaging of neighboring logits can provide a modest smoothing benefit in this setting. The reported K=3 configuration changes only the final post-processing of logits from the trained single-window classifier; it does not modify the training procedure and introduces no additional trainable parameters. The matched fold–seed comparison showed a positive mean change relative to K=1, although the direction of change was not uniform across all runs. This supports the interpretation of K=3 fusion as a useful short-neighborhood post-processing step under the evaluated protocol, rather than as a general temporal-robustness guarantee.

The longer neighborhoods K∈{5,7,9} provide sensitivity information rather than alternative primary configurations. Their results show that using additional neighboring logits can yield small further changes, but the incremental differences become minor at longer neighborhoods. Thus, the analysis supports the presence of a limited temporal-smoothing effect, while not establishing an optimal neighborhood length. Because the fusion is centered within completed source recordings and may use subsequent windows, all fused configurations should be interpreted as offline post-processing rather than causal online inference.

### 5.5. Single-Window Class-Wise Error Structure

The class-wise analysis shows that the high aggregate macro-F1 does not imply identical behavior across UAV types. Under the K=1 single-window reference, the main off-diagonal confusion patterns involved DJI Phantom predicted as DJI M600 or as WL F949s, and DJI Mavic 2 predicted as DJI Phantom. These class-pair patterns identify where the remaining single-window errors are concentrated and provide useful diagnostic targets for future data collection or condition-controlled analysis.

The current experiment does not isolate the physical causes of these confusions. Factors such as rotor configuration, target aspect, nominal range, signal-to-noise ratio, acquisition session, or background conditions may affect micro-Doppler appearance, but this study does not include controlled interventions to assign a specific cause to each confusion pair. The rotor-count annotation in Table 2 assigns four rotors to DJI Mavic 2, DJI Phantom, and DJI Inspire 2. For the confusion pairs among these three platforms (DJI Mavic 2 predicted as DJI Phantom at 1.78%, DJI Phantom predicted as DJI Inspire 2 at 0.52%, and DJI Inspire 2 predicted as DJI Phantom at 0.37%), comparable blade-passing periodicities provide a plausible physical account of the residual time–frequency overlap [4,6]. The largest off-diagonal entry, DJI Phantom predicted as DJI M600 at 4.07%, cannot be explained by rotor-count similarity, since the two classes are annotated with four and six rotors, respectively. Rotor-count-independent factors, such as the overall Doppler spread of the platform, echo strength and signal-to-noise ratio, or dominant fuselage returns, may instead contribute to this confusion; the comparatively high intra-class variability of DJI Phantom (mean class F1 95.05±3.05%, the lowest among the six classes) is also consistent with a fraction of its windows falling into the DJI M600 feature region. For DJI Phantom predicted as WL F949s at 2.74%, the dataset annotation assigns WL F949s a rotor count of 1, so its micro-Doppler signature is expected to be dominated by platform bulk motion rather than multirotor blade modulation; the observed confusion suggests that a minority of windows carry insufficient discriminative cues, for example short dwell time, low signal-to-noise ratio, or similar radial-velocity structure. These interpretations remain hypotheses rather than established causes, because the experiment includes no controlled interventions and does not measure rotor rate or blade geometry directly. In addition, the pooled confusion matrix combines repeated model decisions from multiple seeds on the same fold-specific held-out recordings. It should therefore be read as a descriptive summary of model behavior under the evaluated protocol, not as independent recording-level evidence.

### 5.6. Limitations

- System boundary. The evaluated pipeline begins from a range-localized target return. Automatic target detection, target-presence estimation, automatic range localization, multi-target association, and range-bin tracking are outside the evaluated scope. The reported classification results therefore do not include errors from those upstream stages.
- Dataset and acquisition boundary. Evaluation is limited to the balanced three-recording-per-class subset selected from LSS-FMCWR-1.0. Recording-disjoint splitting removes direct source-recording overlap within each fold, but acquisition-condition confounding may remain. Additional experiments would be needed to assess cross-radar, cross-site, cross-range, cross-session, or open-environment generalization.
- Dependence of evaluation windows. Windows extracted from the same source recording are temporally correlated. The nine fold–seed outcomes summarize three recording-disjoint folds evaluated with three model seeds, but they are not nine independent datasets. The reported standard deviations describe fold–seed variability and are not confidence intervals.
- Closed-set scope. The evaluated task contains six known UAV classes. Unknown UAV types, non-UAV targets, clutter-only inputs, and explicit rejection decisions are not evaluated. Open-set recognition and target-screening behavior therefore remain outside the present scope.
- Observation-interface scope. The proposed three-channel representation is compared with a repeated-channel reference, and per-channel attribution is examined through a seven-configuration ablation that zeroes individual channels while keeping the three-channel stem, classifier architecture, and training hyper-parameters unchanged (Table 5). What the study does not perform is a systematic representation search or a comparison against a broad set of alternative time–frequency inputs; moreover, the observed per-channel differences are small relative to fold–seed variability and are reported descriptively rather than as significance claims.
- Classifier-comparison scope. The compact depthwise CNN is used as a fixed classifier for controlled evaluation. The study does not compare multiple backbone families, pretrained image models, transformer-based architectures, or larger radar-specific networks. Transfer learning from models pretrained on generic optical imagery is a recognized alternative, but the domain gap between natural images and radar micro-Doppler spectrograms limits direct transfer; input normalization and channel adaptation are typically required to reconcile differing intensity statistics and channel semantics [33]. In the small-sample regime considered here, transfer-based initialization or fine-tuning has been shown to benefit micro-Doppler classification when training support is limited [34]. Such pretraining would, however, introduce acquisition- and source-dependent factors that differ from the controlled scratch-trained setting of this study, and is therefore left to future work.
- Training-protocol scope. All models are trained with a fixed 20-epoch budget without a validation split or early stopping. The fixed budget keeps training identical across the compared configurations, but validation-based schedules with early stopping on held-out validation data remain a natural alternative and are left for future evaluation. The learning-rate scheduler therefore also monitors a training-side signal, which is a direct consequence of this design rather than a validation-based schedule.
- Temporal scope. The reported K=3 fusion and the longer-neighborhood sensitivity settings use centered within-recording neighborhoods and may include subsequent windows. They therefore characterize offline post-processing rather than online, real-time, or causal sequential operation. The reported K=3 setting is a short-neighborhood configuration, not an empirically optimized temporal length.

## 6. Conclusions

This study evaluated a deterministic three-channel micro-Doppler observation interface and offline decision-stage temporal logit fusion for closed-set six-class post-localization FMCW radar UAV classification. Using a compact scratch-trained depthwise CNN with 52,918 trainable parameters, the proposed approach was assessed under a recording-disjoint protocol with three held-out-recording folds and three model seeds.

The component ablation showed that mean macro-F1 changed from 97.67% with repeated-channel input to 98.35% with the proposed three-channel K=1 single-window configuration under the same classifier architecture and evaluation grid. The proposed K=1 single-window reference achieved 98.36±0.89% accuracy and 98.35±0.90% macro-F1 across the nine fold–seed runs. These results support the use of the deterministic three-channel observation interface as the main single-window representation in the evaluated protocol. A seven-configuration per-channel ablation further showed that no single channel accounts for this gain, although the per-channel differences are small relative to fold–seed variability.

As the reported offline temporal post-processing stage, the centered within-recording K=3 logit-fusion configuration achieved 98.62±1.02% accuracy and 98.60±1.04% macro-F1. This corresponds to a paired descriptive macro-F1 change of +0.26±0.33 percentage points relative to the matched K=1 single-window reference. Longer neighborhoods with K∈{5,7,9} were included only as temporal-neighborhood sensitivity settings; they indicated small additional changes but did not establish an optimal neighborhood length.

The class-wise analysis showed that the remaining single-window errors were not uniformly distributed across UAV types, with the main confusion patterns involving DJI Phantom predicted as DJI M600 or as WL F949s, and DJI Mavic 2 predicted as DJI Phantom. These results provide diagnostic information for future condition-controlled data collection and analysis.

An expanded-subset evaluation on recordings with varied modulation periods, frequency bands, and nominal ranges showed a mean decrease of about 1.5 percentage points relative to the main subset, concentrated in a single fold whose held-out DJI Mavic 2 recording was frequently confused with DJI Phantom. This provides initial evidence that the same architecture and protocol can be applied beyond the single radar configuration of the main experiment, without establishing quantitative generalization guarantees across radar configurations.

The conclusions are limited to the evaluated recording-disjoint, post-localization, closed-set six-class protocol. Beyond this initial configuration-sensitivity evidence, further evaluation would be required to assess automatic target detection and range localization, open-set recognition, cross-radar, cross-site, cross-range, cross-session, or open-environment generalization, and hardware-specific or end-to-end sensing latency.

## Figures and Tables

**Figure 1:**
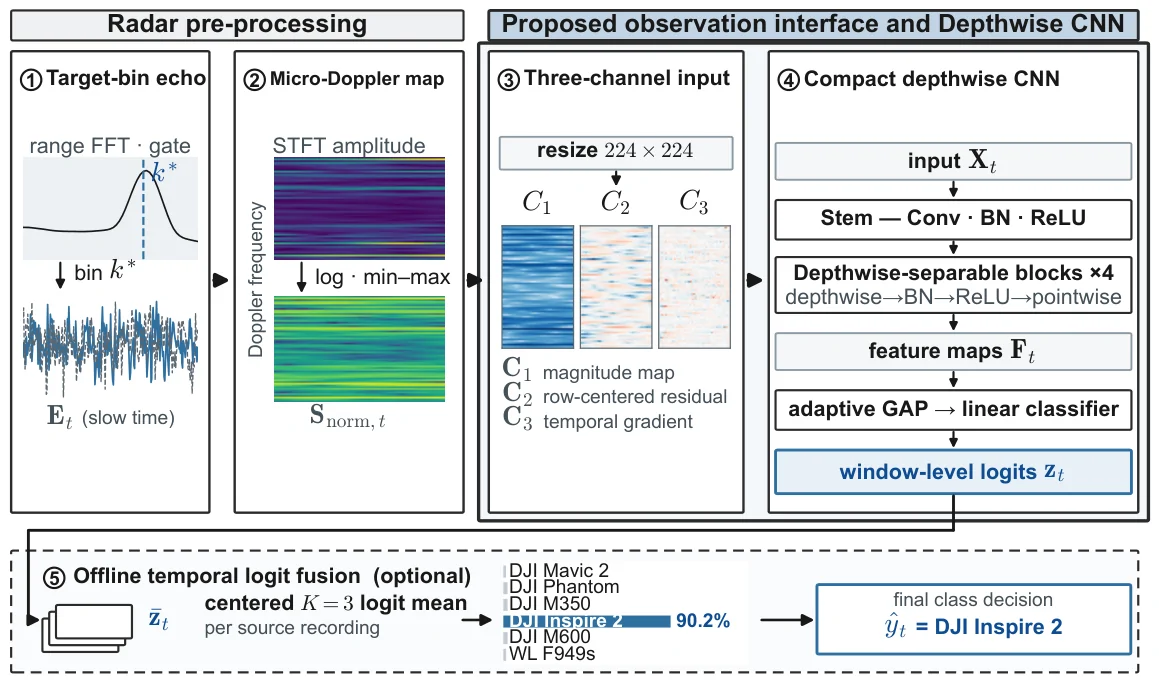
Post-localization classification pipeline. A fixed three-channel micro-Doppler observation is classified by the compact depthwise CNN. Optional offline temporal logit fusion averages window-level logits within each source recording before the final class decision. The class-probability bars illustrate a single fused example window (recording-disjoint Fold 1, model seed 0); the printed percentage is the winning-class fused confidence of that window, not an aggregate accuracy.

**Figure 2:**
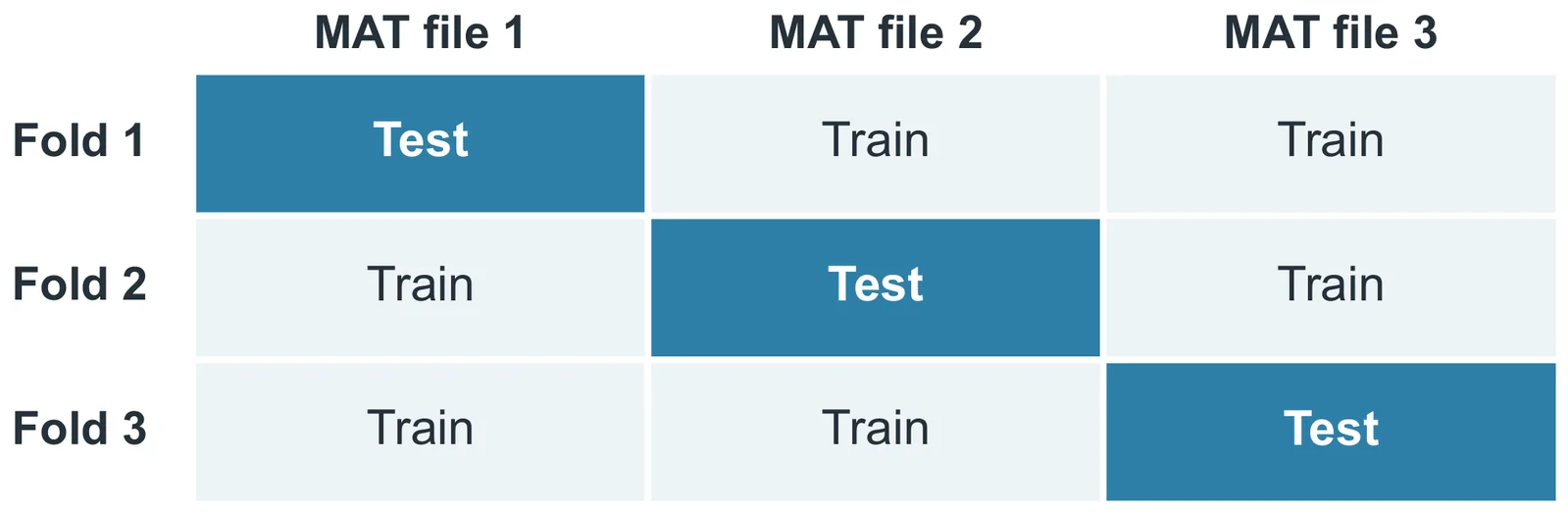
Recording-disjoint leave-one-recording-out protocol. For each UAV class, one of three selected source MAT recordings is held out for testing in each fold, while the remaining two recordings provide training windows. Source recordings are not shared between training and testing within a fold.

**Figure 3:**
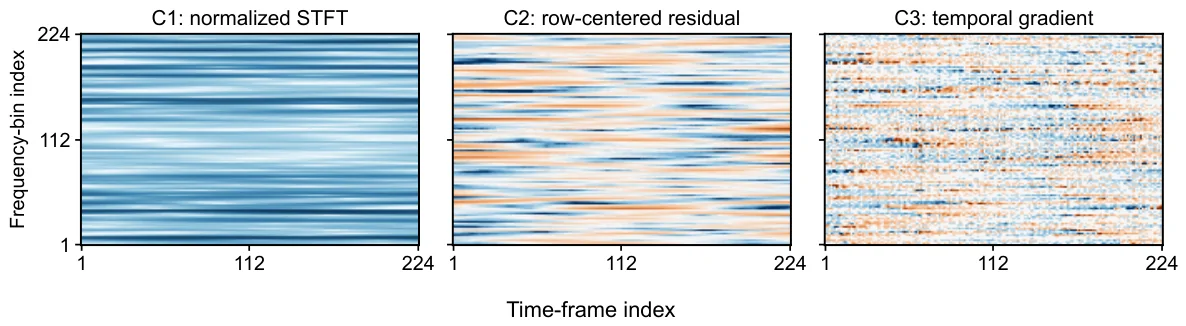
Example deterministic three-channel micro-Doppler observation. C1 is the normalized magnitude map, C2 is the signed row-centered residual, and C3 is the signed temporal gradient; C1 is linearly mapped to [−1,1] at the classifier input.

**Figure 4:**
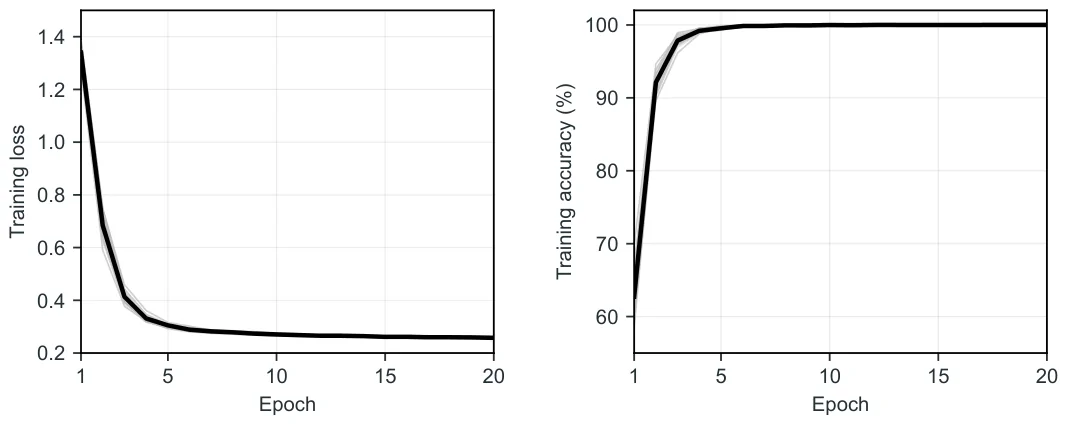
Training convergence of the nine main-experiment fold–seed runs. (**Left**): Training loss per epoch. (**Right**): Training accuracy per epoch. Light gray curves show the nine individual runs; the black curve shows the mean and the shaded band the sample standard deviation across the nine runs.

**Figure 5:**
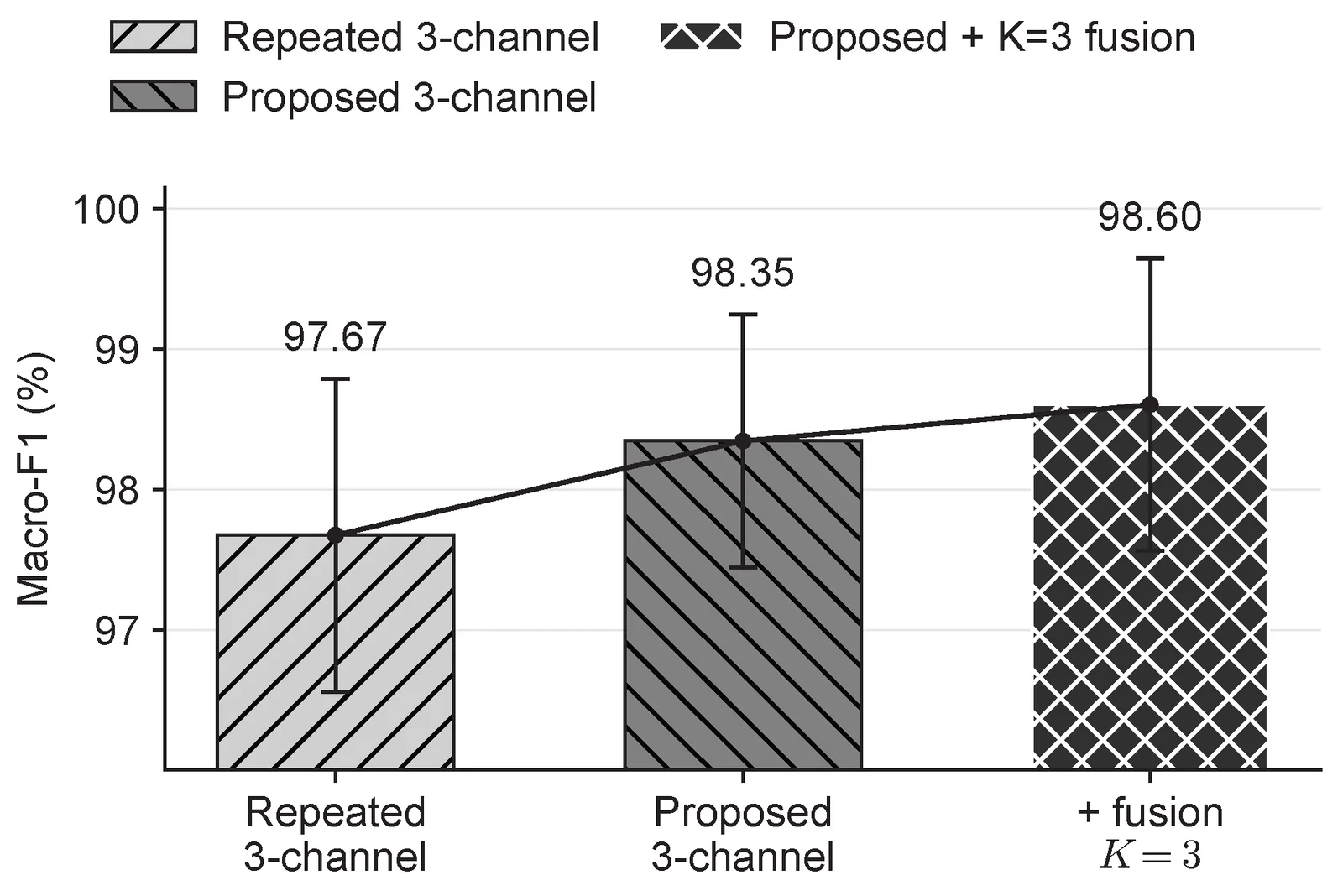
Component contribution ablation. Bars show mean macro-F1 and error bars show sample standard deviation across the nine fold–seed runs. The repeated-channel reference uses the same magnitude-derived input plane in all three channels; the proposed setting uses the deterministic three-channel observation; and the final setting adds K=3 offline temporal logit fusion.

**Figure 6:**
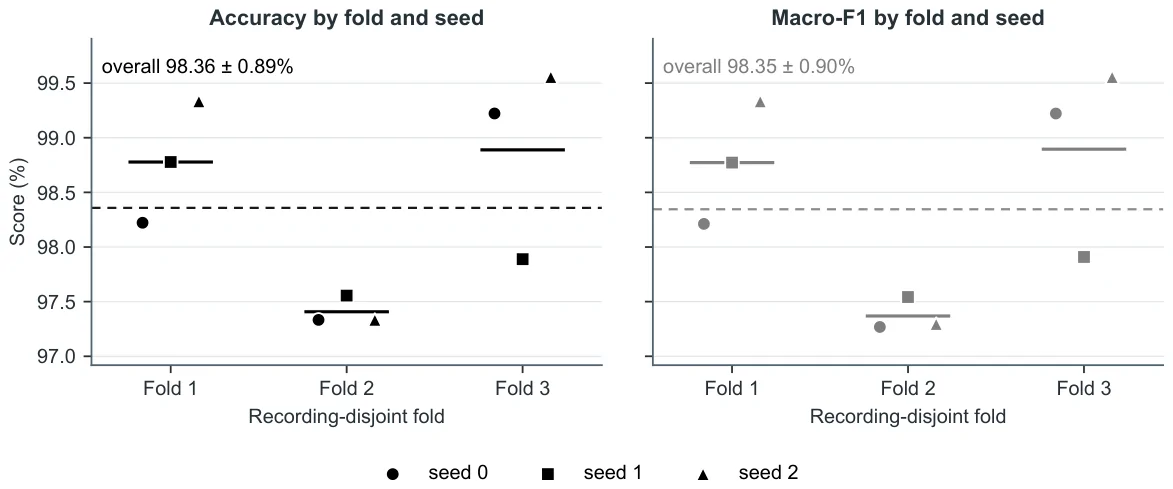
K=1 single-window fold–seed performance. Markers show accuracy and macro-F1 for model seeds 0–2 within each recording-disjoint fold. Overall values are summarized as mean ± sample standard deviation across the nine fold–seed runs.

**Figure 7:**
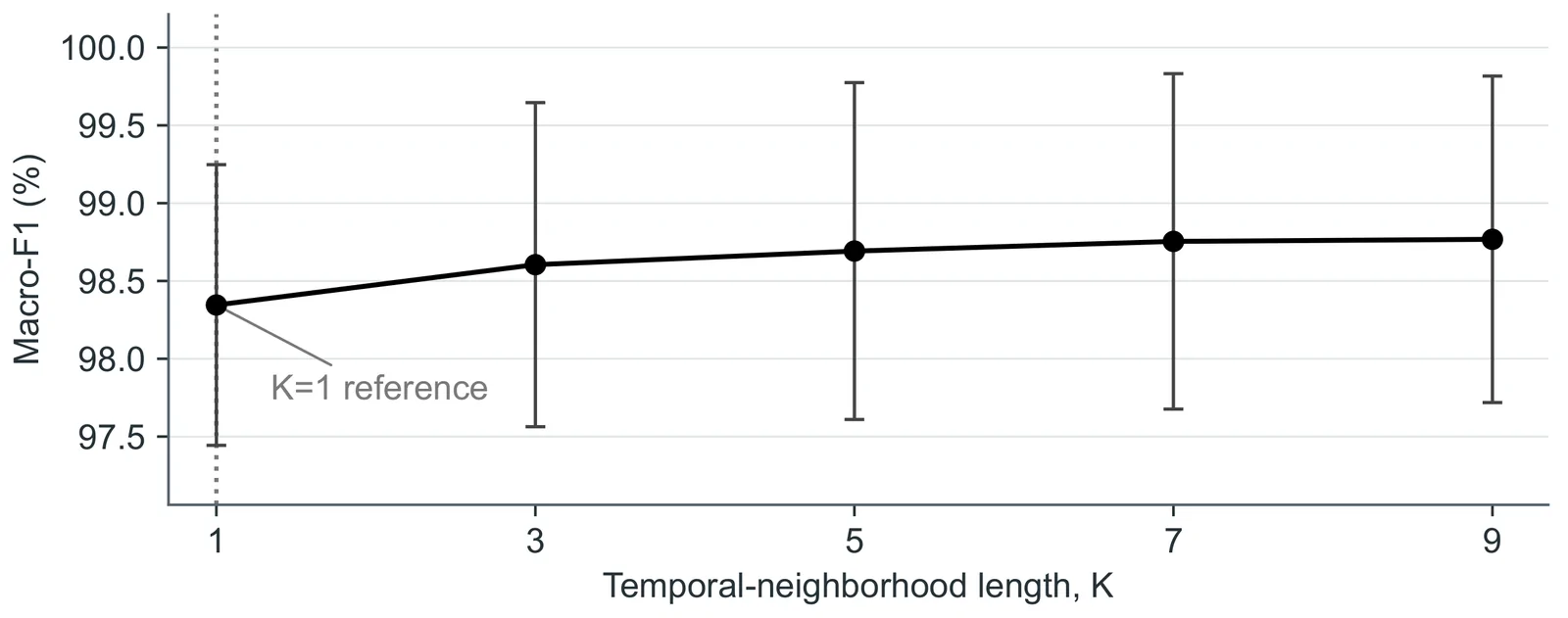
Temporal-neighborhood sensitivity. Points show mean macro-F1 and error bars show sample standard deviation across the nine fold–seed runs. For K>1, the trained classifier and window-level logits are unchanged; only the temporal-fusion length is varied.

**Figure 8:**
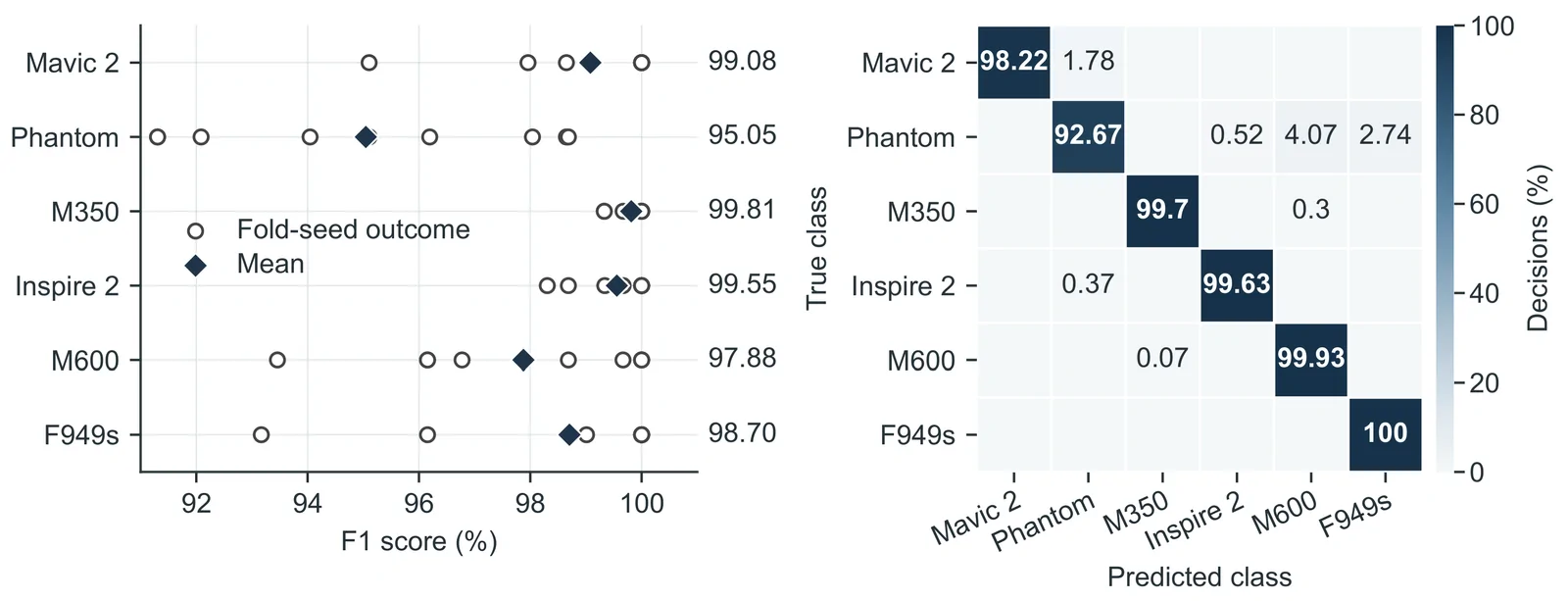
K=1 single-window class-wise performance and pooled confusion pattern. (**Left**): Fold–seed class-wise F1 outcomes with class means. (**Right**): Pooled row-normalized confusion matrix obtained by summing the nine K=1 run-level confusion-count matrices before row normalization.

**Table 1:** Representative related work reviewed in Section 2.1, Section 2.2, Section 2.3, Section 2.4 and Section 2.5, organized by input representation and classifier category. det.: deterministic; scratch: scratch-trained.

Reference	Input Representation	Classifier
Single-representation, standard classifiers		
[8]	Micro-Doppler sig.	SVM
[9]	Spectrogram	CNN
[10]	Micro-Doppler	Analysis
[22]	Micro-Doppler	Sparse repr.
[11]	Micro-Doppler img.	CNN
Input representation studies		
[27]	Micro-Doppler repr.	DL comparison
[28]	Time–frequency	ETF-MDNet
[29]	Input format study	CNN
Compact CNN classifiers		
[13]	Micro-Doppler	Lightweight CNN
[14]	Micro-Doppler	ECM Net
[15]	Micro-Doppler	CT-RURnet
This study	3-channel (det.)	DwCNN (scratch)

**Table 2:** Acquisition details of the selected LSS-FMCWR-1.0 recordings used in this study. The “Rotors” column reports the rotor count assigned to each class by the dataset annotation. Each row summarizes the three same-configuration source recordings (trials 1–3) selected for the corresponding class, giving 18 source recordings in total.

Class ID	UAV Type	Date	Period (ms)	BW (MHz)	Range (m)	Rotors
0	DJI Mavic 2	8 May 2023	0.3	100	9	4
1	DJI Phantom	8 May 2023	0.3	100	9	4
2	DJI M350	2 August 2023	0.3	100	12	4
3	DJI Inspire 2	8 May 2023	0.3	100	11	4
4	DJI M600	8 May 2023	0.3	100	8	6
5	WL F949s	1 January 2025	0.3	100	9	1

**Table 3:** Micro-Doppler observation parameters used in all experiments.

Parameter	Setting
Slow-time segment length	256 chirps
STFT window	128-point Hamming window
STFT overlap	127 samples
STFT length	1024 points
Doppler spectrum	Two-sided
Amplitude representation	Log-magnitude
Logarithmic stabilizer $\epsilon_{\log}$	$10^{-8}$
Spectrogram normalization	Per-window min–max scaling
Spatial resizing	Bilinear interpolation
Observation size	224×224

**Table 4:** Architecture of the compact depthwise CNN used for six-class UAV classification.

Stage	Operation	Stride	Output	Parameters
Stem	3×3 Conv + BN + ReLU	2	24×112×112	696
Block 1	DW 3×3 + PW 1×1	2	48×56×56	1512
Block 2	DW 3×3 + PW 1×1	2	96×28×28	5328
Block 3	DW 3×3 + PW 1×1	1	160×28×28	16,736
Block 4	DW 3×3 + PW 1×1	1	160×28×28	27,680
Head	GAP + Dropout + Linear	–	6 logits	966
Total	–	–	–	52,918

**Table 5:** Per-channel ablation of the three-channel observation interface. Each configuration zeroes the listed channel(s) at the input stage while keeping the three-channel stem and the classifier unchanged. Values are mean ± sample standard deviation across the nine fold–seed runs.

Input Channels	Accuracy (%)	Macro-P (%)	Macro-F1 (%)
C1 + C2 + C3 (full)	98.36 ± 0.89	98.50 ± 0.78	98.35 ± 0.90
C1 + C2	98.06 ± 0.93	98.25 ± 0.77	98.05 ± 0.94
C1 + C3	98.09 ± 0.85	98.23 ± 0.78	98.07 ± 0.88
C2 + C3	97.57 ± 1.18	97.72 ± 1.02	97.53 ± 1.23
C1 only	97.89 ± 0.57	98.06 ± 0.53	97.87 ± 0.60
C2 only	94.11 ± 2.52	94.73 ± 2.09	93.98 ± 2.64
C3 only	98.01 ± 1.11	98.13 ± 1.03	98.00 ± 1.12

Macro-recall equals accuracy on the class-balanced test sets and is omitted.

**Table 6:** Single-window reference and reported temporally fused performance. Values are mean ± sample standard deviation across the nine fold–seed runs. Paired ΔMacro-F1 is computed within matched fold–seed pairs relative to K=1 and is reported in percentage points (pp).

Configuration	Accuracy (%)	Macro-F1 (%)	Paired ΔMacro-F1 (pp)
K=1 single-window	98.36±0.89	98.35±0.90	0.00±0.00
K=3 reported fusion	98.62±1.02	98.60±1.04	+0.26±0.33

**Table 7:** Sensitivity to the offline centered within-recording temporal neighborhood length. Macro-F1 is reported as mean ± sample standard deviation across the nine fold–seed runs. Paired ΔMacro-F1 is computed within matched fold–seed pairs relative to K=1. W/T/L denotes the number of positive, unchanged, and negative paired changes, respectively.

*K*	Role	Macro-F1 (%)	Paired ΔMacro-F1 (pp)	W/T/L
1	Single-window reference	98.35±0.90	0.00±0.00	0/9/0
3	Reported fusion	98.60±1.04	+0.26±0.33	6/1/2
5	Sensitivity	98.69±1.08	+0.35±0.36	8/0/1
7	Sensitivity	98.75±1.08	+0.41±0.44	7/2/0
9	Sensitivity	98.77±1.05	+0.42±0.46	8/1/0

**Table 8:** Radar acquisition parameters of the expanded-subset recordings, parsed from the 18 selected source filenames. The main-experiment configuration is 0.3 ms modulation period, 100 MHz bandwidth, and K-band; the last column indicates which parameters differ from it.

UAV Class	Period (ms)	BW (MHz)	Band	Range (m)	Changed vs. Main
DJI Mavic 2	1.024	100	L	9	Period, band
DJI Phantom	1.024	100	K	6	Period
DJI M350	1.024	100	L	12	Period, band
DJI Inspire 2	1.024	100	L	11	Period, band
DJI M600	1.024	100	L	13	Period, band
WL F949s	0.3	100	L	8	Band

WL F949s recordings are stored in a dedicated frontal-view low-speed subdirectory of the dataset tree.

**Table 9:** Expanded-subset evaluation. The same depthwise CNN three-channel configuration was evaluated on recordings with varied radar acquisition parameters. Values are mean ± sample standard deviation; each per-fold row aggregates its three seeds and the pooled row aggregates all nine fold–seed runs.

Fold	Macro-F1 (%)	Accuracy (%)
1	92.76 ± 1.86	93.04 ± 1.74
2	99.67 ± 0.22	99.67 ± 0.22
3	98.03 ± 0.82	98.04 ± 0.82
Pooled (mean ± std, n=9)	96.82 ± 3.29	96.91 ± 3.14

## Data Availability

No new radar recordings were collected in this study. The raw radar recordings analyzed in this study are publicly available through the official *Journal of Radars* data page for the LSS-FMCWR-1.0 multiband FMCW radar low-altitude target detection dataset, available at https://radars.ac.cn/web/data/getData?newsColumnId=62117dab-1688-4a5e-834d-646b09724520 (accessed on 15 July 2025), and are described in Ref. [24].

## Abbreviations

The following abbreviations are used in this manuscript:

BN	Batch normalization
CNN	Convolutional neural network
CUDA	Compute Unified Device Architecture
DW	Depthwise
FFT	Fast Fourier transform
FMCW	Frequency-modulated continuous-wave
GAP	Global average pooling
MAT	MATLAB MAT-file
PW	Pointwise
STFT	Short-time Fourier transform
UAV	Unmanned aerial vehicle
